# Quality of Drinking Water from Springs in Palestine: West Bank as a Case Study

**DOI:** 10.1155/2019/8631732

**Published:** 2019-06-23

**Authors:** Azza Daghara, Issam A. Al-Khatib, Maher Al-Jabari

**Affiliations:** ^1^Faculty of Graduate Studies, Birzeit University, Birzeit, State of Palestine; ^2^Institute of Environmental and Water Studies, Birzeit University, Birzeit, State of Palestine; ^3^Mechanical Engineering Department, Faculty of Engineering and Technology, Palestine Polytechnic University, Hebron, West Bank, State of Palestine

## Abstract

The shortage of fresh water creates acute challenges in the West Bank of Palestine. Springs provide a main water resource in the West Bank. Investigating springs' water quality is essential step for promoting their public use. The aim of this research is to assess the microbiological and physiochemical quality parameters of drinking water from springs. The study methodology included sampling through field work and laboratory testing for water quality parameters using standard procedures. The study area covered all locations containing licensed springs by the Palestinian Water Authority in the West Bank of Palestine. The number of collected samples was 127 covering 300 springs. The chemical, physical, and biological parameters for each sample were measured. Then, the obtained characteristics were evaluated based on national and international quality standards (PSI and WHO). The investigated parameters included temperature, pH, EC, total hardness, concentrations of nitrate, sodium ions, total chlorine, residual chlorine, turbidity, and total and faecal coliforms. Most of investigated physical and chemical parameters were within the acceptable standard limits. However, the turbidity and chloride and nitrate concentrations exceeded standard limits. The findings indicate that only a minor fraction of the samples (2%) requires chlorination treatment, while most of the springs (97% of samples) are classified as possessing no risk.

## 1. Introduction

The climate of Palestine is arid and semiarid. Water resources in Palestine are limited. Groundwater is the main water source in the West Bank. Previous studies have shown that some areas in the West Bank, especially rural areas, rely on other sources of water such as collecting rainwater in the rain-fed cisterns. In fact, water supply is one of the most critical issues in the Middle East. Water shortage and water quality are imminent [1, 2]. Water is viewed as a standout amongst the most imperative and delicate issues in the Middle East [3]. The Jordan River is the main water source that can be utilized as a surface water asset for the West Bank. However, it is controlled by Israel. The surface water assets are rare and constrained [4]. Thus, the groundwater is the main water supply for the Palestinian individuals [4]. There are three groundwater aquifer bowls in Palestine. They are located under the West Bank, but controlled by Israel.

The WHO has set the minimum per capita daily consumption of 100 liters per day [5]. However, in Nablus and Hebron cities of the West Bank, the daily consumption is about 50 liters per capita per day. The mean value of the daily consumption in the West Bank is 66 liters per capita per day [3, 6, 7]. Such a clear deficiency of clean fresh water with the increasing demand for industrial, agricultural, and domestic uses is one of the main problems in Palestine [8].

Poor water quality is considered as an indication of poverty. Water quality is influenced by the local practices of waste disposal and wastewater discharge [9]. The toxins can penetrate through soil and rock into the groundwater resources, resulting in physical and substance changes and in changes in water quality [10]. For example, around 70% of the water in India has been turned out to be contaminated because of the release of household sewage and mechanical effluents into regular water sources [11]. There are many factors that lead to water pollution in the West Bank. These include the absence of sewage networks in many rural areas, the reliance on cesspits to collect domestic wastewater, the poor sanitation networks, and the improper waste management and leachate from random waste dumps [1, 12]. In most of the West Bank areas, water quality is not monitored [13].

Water pollution is defined as a change in the composition or conditions of the watercourse components, due to human activity, that makes water becoming less suitable for its natural uses [9]. Water must not contain microbiological contaminations that are pathogenic to humans [14]. Microbiological contamination of drinking water causes numerous irresistible ailments, such as cholera, enteric fever, hepatitis, and looseness of the bowels [15, 16]. For noncleaned water supplies, the permission limits reach up to 10 faecal coliforms (FC) per 100 mL [12].

Water quality is indicated by various physical parameters such as pH, total solids, total dissolved solids, total suspended solids, alkalinity, free CO_2_, dissolved oxygen, hardness, chlorine content, and sodium content. Nitrate contamination results from human and animal wastes, soil nitrogen content, plant debris, industrial effluents and chemicals, and seepage and silage through drainage system [17, 18]. The turbidity in the groundwater is an indication of pollution of water resulting from deterioration of organic matter and the improper disposal of domestic and industrial solid wastes and wastewater. Electrical conductivity (EC) is a measure of the presence of dissolved salts in water which are responsible to conduct electric current [19]. Total hardness (mg/l) is defined as the sum of magnesium and calcium carbonate contents. High magnesium content affects the domestic use of water [20]. High levels of water hardness lead to heart diseases and kidney stone formation [21].

Springs are unique ecosystems; however, in many cases, they are severely threatened and thus require an urgent need for better management and conservation [22]. Identifying the chemical, physical, and microbiological characteristics of water is a key step in the management of the springs in the West Bank. Such characteristics are essential in the environmental planning for efficient utilization of these natural resources. They are important parameters in directing the efforts to remove potential sources of contamination from the spring's drainage area and to select and design water purification units [23].

Water deterioration of springs can be attributed to the strong interaction of surface water and groundwater. It is strongly affected by the hydrogeological conditions and the surrounding environment [24]. Thus, there is a need for intensive, long-term monitoring of groundwater level and water quality. This can lead to a better evaluation of the groundwater contamination [25].

Microbial parameters include total coliforms and faecal coliforms [8, 21, 26]. The presence of total coliforms in the water does not indicate water contamination. However, the faecal coliforms found in the groundwater are an indication of contamination from human or animal sewage [27–29]. Contaminated drinking water with high concentration of nitrate causes methemoglobinemia in infants less than 6 months of age.

Deterioration of water quality in Palestine and worldwide is a key environmental challenge that requires urgent action [30, 31]. The aim of this study is to examine the quality of drinking water from springs in the West Bank. The investigated parameters include chemical, physical, and microbiological characteristics for assessing the levels of water pollution in the water springs.

## 2. Research Methodology

A cross-sectional study was conducted for assessing the physicochemical and bacteriological quality of drinking water from springs. It included sampling through field work and analysis of water quality parameters using standard laboratory testing.

### 2.1. Study Area

**Figure 1:**
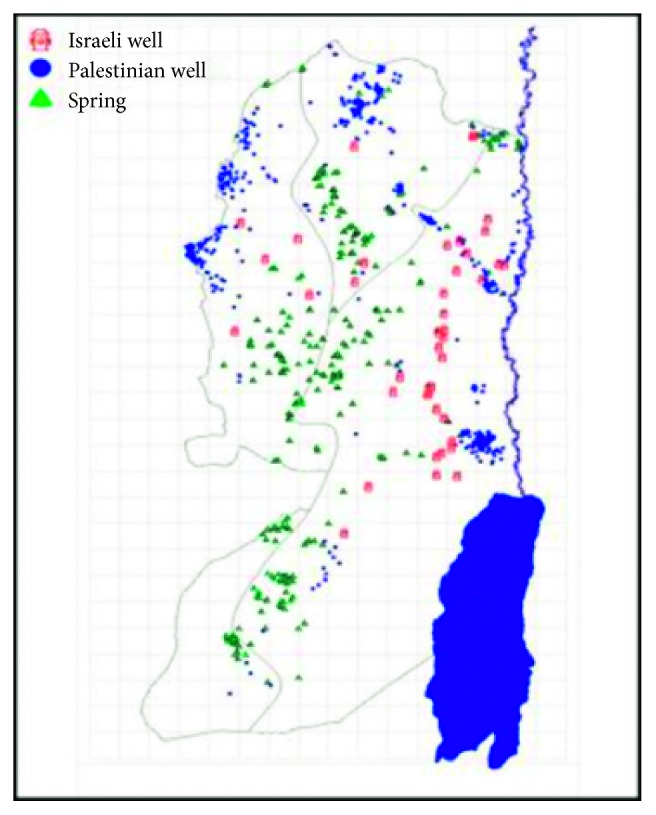
The geographic distribution of the springs in the West Bank.

The study area included most parts of the West Bank in Palestine.  Figure 1 provides the West Bank map and the geographic distribution of the springs covered in this study. The West Bank is a landlocked region close to the Mediterranean shoreline of Western Asia, with an area of 5,655 km^2^ and a population of 3,284,787. Its main part is under the Israeli control or under the joint Israeli-Palestinian Authority. The climate of the West Bank is Mediterranean to a continental atmosphere [32].

### 2.2. Sampling and Data Collection

Water quality data were collected from the official records of PWA in the West Bank. Water samples (127) were collected from 127 springs, selected from 300 springs in the West Bank of Palestine (see Figure 1). The samples were collected by the PWA staff in the year 2016 from the licensed springs for drinking by the PWA.

### 2.3. Water Analysis

For each water sample, the physicochemical and biological characteristics were measured using standard testing procedures [33]. Triple replicates were used in the analysis of each parameter. The investigated parameters included temperature, pH, EC, total hardness, concentrations of nitrate, sodium ions, total chlorine, residual chlorine, turbidity, and faecal and total coliforms. The temperature, pH, EC, and turbidity parameters were examined in situ, using a thermometer, a portable digital pH meter, an EC meter, and a turbidity meter according to the standard procedures [33]. For all laboratory tests, the samples were stored in 1000 ml sterile glass bottles and then sent to the laboratory of PWA on the same day of collection. The samples were preserved in the refrigerator until testing time according to standard water testing practice [12, 33, 34]. The remaining physicochemical parameters were tested using standard methods using DR 2400 spectrophotometer [33]. Total and faecal coliforms counts were measured by the membrane filtration technique [33].

The obtained data were recorded and categorized in tables as Microsoft Excel spreadsheets for further analysis. The obtained water characteristics were then compared to national and international quality standards, including the Palestine Standards Institution (PSI) and the WHO. Then, risk analysis was performed according to the obtained range of total coliforms and faecal coliforms.

## 3. Results and Discussions

### 3.1. Physicochemical Characteristics

Table 1 lists the obtained analytical results of the physicochemical parameters. It lists the ranges and the mean values of the tested water quality characteristics of the studied springs. It also lists the related permission limits according to the PSI and WHO for comparison.

### 3.2. Water Temperature

The measured temperature ranged from 18°C to 27°C, with a mean vale of 23°C. The maximum temperature was recorded in the period of June and September while the minimum temperature was recorded in January. This is a typical temperature range within the Mediterranean climate. Water temperature may affect water quality through biological activities. However, the obtained temperature range is normal and thus possesses no risk on water quality.

### 3.3. pH

Water from springs has nearly neutral to slightly alkaline characteristics, with a narrow range of 7.08 to 8.19. The maximum pH value was recorded in May while the minimum value was recorded in October. These variations could be due to geological and seasonal variations in the alkalinity of surrounding areas to springs sources. These results are within the permission limit of the WHO and PSI standards. Consequently, water quality related to pH is acceptable.

### 3.4. Turbidity

The obtained turbidity was within the range of 0.05–9.9 NTU, with a mean value of 1.57 NTU. A small fraction of the samples (25%) was found to have turbidity values above the permission limit. The maximum value was recorded in March. This might be due to human activities, a decrease in the water level, and an increase in the suspended particulate matter. Five samples were found to have turbidity above the permission limits set by the WHO and PSI guidelines (5.0 NTU). This indicates that springs are contaminated with suspended materials and natural colloids such as silt and clays. This may be attributed to interaction of springs with surface water, especially during heavy rains or spring runoff [35, 36]. These results have a wider range than that reported in a previous study of Daghrah [23] for water quality of Wadi Al Qilt springs in the West Bank between November 2004 and March 2007. The previous reported range of turbidity was 0–2 and was within the PSI and WHO allowable limits.

### 3.5. Electrical Conductivity (EC)

The values of electrical conductivity ranged from 473–1406 *μ*S/cm with a mean value of 764 *μ*S/cm. These variations are attributed to differences in geological structures, agricultural activity, and soil conditions within the study area. The observed wide range of EC is an indication of high content of dissolved salts such as sodium chloride and potassium chloride. A considerable part of the samples (41%) exceeded EC permission limit. Usually, groundwater tends to have a high electric conductivity, resulting from the presence of high amounts of dissolved inorganic substances in ionized form. According to Marmontel et al. [37], the significant variation in EC values can be attributed to the different land uses, the spatial variation of spring locations, and the state of conservation of the vegetation. The electrical conductivity is an important parameter in determining the suitability of water for a specific purpose. Water quality is classified according to the range of EC as shown in Table 2 [26]. Obviously, water from springs in this case study is classified as good or permissible. All measured values of EC were within the permission limits of the WHO and PSI standards (2000 *μ*Scm^−1^).

### 3.6. Chloride Content

Chloride content ranged from 22.0 to 284 mg/L, with a mean value of 75.4 mg/L. The maximum value (284 mg/L) was recorded in April, while the minimum value (22.0 mg/L) was recorded in May. The maximum value exceeded the permission limit (250 mg/L) according to the PSI and WHO. A small part of samples (24%) had chloride content above the permission limit.

### 3.7. Nitrate Content

The measured nitrate concentration ranged from 0 to 106 mg/L, with a mean value of 30 mg/L. A small fraction of samples (21%) was found to have a nitrate concentration above the permission limit. Fifty water samples were found to have nitrate concentration exceeding the permission limits set by the WHO and PSI (10 mg/l). Our results indicate that some groundwater resources are contaminated with nitrate. This is expected to be resulting from the penetration of nitrates from sewage and other wastes. Contaminated water with nitrates is not suitable for domestic uses, since it causes diseases and health problems to humans and animals. These findings are higher than the previously reported range of the nitrate content. Daghrah [23] reported that the nitrate content for water from Wadi Al Qilt springs in the West Bank between November 2004 and March 2007 varied between 17 and 45 (mg·NO_3_-N/L).

### 3.8. Total Hardness

The total hardness ranged from 199 to 485 mg/L, with a mean value of 345 mg/L. The maximum value of total hardness of 485 mg/L was recorded in May, while the minimum value of 199 mg/L was found in February. A small fraction of the samples (about 10%) was found to have a hardness value above the permission limit of the PSI. Water quality can be classified according to total hardness as indicated in Table 3 [19]. The classification of the obtained values of the total hardness for water from springs ranges from hard to very hard.

### 3.9. Sodium Content

The sodium ion concentration ranged from 16.9 to 137 mg/L, with a mean value of 41 mg/L. Only a small fraction of the samples (15%) was found to have a sodium content exceeding the permission limit of the WHO. This indicates that there is a little contamination of groundwater resources with sodium ions in the West Bank springs. The obtained range is wider than that previously reported by Daghrah [23] for water from Wadi Al Qilt springs in the West Bank between November 2004 and March 2007 (15–31 mg/L).

### 3.10. Microbiological Characteristics

Table 4 lists the obtained analytical results of the microbiological parameters. It lists the results of water quality characteristics of springs in this case study. It also lists the related permission limits according to the PSI and WHO. The measured values of total coliforms ranged from 0 to 27 CFU/100 mL. A very small fraction of the samples (4%) was found to have total coliforms higher than the acceptable limit. The maximum value of total coliforms was recorded in September, while the small values were recorded in various months. Results of faecal coliforms indicated that most of the groundwater has no faecal coliforms. Only a very small fraction of the samples (3%) was contaminated with faecal coliforms. This level of contamination with faecal coliforms is lower than that reported in previous work of Daghrah [23] who found that 47% of the springs' samples were contaminated with faecal coliforms.

These results indicate that a small percentage of the springs is contaminated with microbiological contents, with values beyond the permission limits set by the WHO and PSI. This is expected to be resulted from the infiltration of sewage and contaminated water through connection and leakage points, back siphoning, seepage framework, and brokenness into water supply networks. The contamination of drinking water with microorganisms causes mortalities and morbidities, due to waterborne illnesses like typhoid, cholera, diarrhea, and hepatitis, as well as protozoan and helminth contaminations.

These results indicate that water treatment is essential before use for the contaminated springs.  Table 5 lists the required treatment procedures recommended by the WHO [3] for the categorized degree of contamination, according to the range of total coliforms. It lists the distribution of tested spring samples for total coliforms according to their level of contamination and the required treatment procedure [3]. Clearly, only 2% of samples with a range of 0–3 CFU/100 mL are categorized with zero degree of contamination and thus do not require any treatment. On the other hand, 2% of the samples with values between 4 and 50 CFU/100 mL are categorized with the first degree of contamination and thus required chlorination treatment only. The remaining percentage (96%) of samples was not contaminated by total coliforms.

Results of risk analysis of water samples are shown in Table 6. It lists the degree of risk and the percentage of tested cistern samples for faecal coliforms (CFU/100 ml) according to classified degree of risk (according to the WHO [3]).

Obviously, 97% of samples possess no risks, while 2% of samples possess a simple risk level. Only 1% of samples possess a moderate risk. However, there is no high risk or very high risk classification in all investigated springs.

## 4. Conclusions and Recommendations

Most of the investigated physical and chemical characteristics for water from springs were within the acceptable standard limits, except turbidity and chloride and nitrate concentrations. Nitrate analysis indicated that part of the samples exceeded the PSI and WHO permission limits (exceeding 10 mg/L). A quarter of the samples was found to exceed the permission limits for turbidity and chloride content. However, the biological contamination was limited. The major part of the samples (97%) was classified to possess no risk, while 2% of the samples were classified to possess simple risk and thus required chlorination treatment.

It is recommended that the PWA should consider the utilization of groundwater from springs as one of the drinking water resources. This requires raising the public awareness on proper utilization and precautions for maintaining water quality, prevented from contaminations by physical, chemical, or biological pollution. Such utilization requires better management of the springs in the West Bank. It is necessary to remove potential sources of contamination from the spring's drainage area. Surface water draining into that area should be redirected away from springs. Most springs will require a continuous disinfection system so that the water is maintained safe for human consumption. A continuous monitoring system is needed through a state-certified water testing laboratory for water quality analysis.

## Figures and Tables

**Table 1:** Physiochemical characteristics of drinking water from licensed spring water in the West Bank compared with standard permission limits of the PSI and WHO.

Characteristics	Unit	Range of measured value	Mean value	Percentage above acceptable limit (%)	Permission limits according to	
					PSI	WHO
Temperature	°C	18–27	23		NA	NA
Turbidity	NTU	0.05–9.9	1.57	25	Up to 5.0	Up to 5.0
Chloride content	mg/L	22.0–284	75.4	24	Up to 250	Up to 250
pH		7.08–8.19	7.55	24	6.5–8.5	6.5–8.5
Electrical conductivity (EC)	*μ*Scm^−1^	473–1406	764	41	Up to 2000	Up to 2000
Nitrates	mg·NO_3_-N/L	0–106	30	21	Up to 10	Up to 10
Total hardness		199–485	344	10	500	NA
Residual chlorine		0–1.39	0.452	16	NA	NA
Total chlorine		0.01–1.69	0.519	18	NA	NA
Na^+^ concentration	mg/L	16.9–137	40.9	15	NA	Up to 200

**Table 2:** Water quality classification for various ranges of EC in *μ*S/cm at 25°C [26].

Range of electrical conductivity (EC) in *μ*S/cm	Water quality classification
<250	Excellent
250–750	Good
750–2,000	Permissible
2,000–3,000	Doubtful
>3,000	Unsuitable

**Table 3:** Water quality classification for various ranges of hardness [19].

Total hardness in mg/L	Degree of hardness
0–75	Soft
75–150	Moderately hard
150–300	Hard
>300	Very hard

**Table 4:** Microbiological characteristics of drinking water from licensed spring water in the West Bank compared with standard permission limits of the PSI and WHO.

Characteristics	Unit	Range of measured value	Percentage above acceptable limit	Permission limits according to	
				PSI	WHO
Total coliforms	CFU/100 mL	0–27	4	0–3	0
Faecal coliforms	CFU/100 mL	0–14	3	0	0

**Table 5:** Distribution of the tested cistern samples for total coliforms according to their level of contamination and required treatment procedure.

Recommended treatment procedure [3]	Range of total coliforms (CFU/100 mL)	Degree of contamination	Contaminated samples (%)
No treatment required	0–3	0	2
Chlorination only	4–50	1	2
Flocculation, sedimentation, and then chlorination	51–50,000	2	0
Very high contamination, need special treatment	<50,000	3	0

**Table 6:** Distribution of tested cistern samples for faecal coliforms (CFU/100 ml) according to classified degree of risk [3].

Range of faecal coliforms (CFU/100 mL)	Degree of risk	Percentage of samples (%)
Zero	No risk	97
1–10	Simple risk	2
11–100	Moderate risk	1
101–1,000	High risk	0
<1000	Very high risk	0

## Data Availability

The data used to support the findings of this study are available from the corresponding author upon request.
